# Emergent surgical intervention for a neonate with premature constriction of the ductus arteriosus with ductal-dependent tricuspid atresia: a case report

**DOI:** 10.1093/ehjcr/ytae640

**Published:** 2024-11-29

**Authors:** Taro Kono, Naofumi F Sumitomo, Hiroyuki Yamagishi, Naritaka Kimura

**Affiliations:** Department of Pediatrics, Keio University School of Medicine, 35 Shinanomachi, Shinjuku-ku, Tokyo 160-8582, Japan; Department of Pediatrics, Keio University School of Medicine, 35 Shinanomachi, Shinjuku-ku, Tokyo 160-8582, Japan; Department of Pediatrics, Keio University School of Medicine, 35 Shinanomachi, Shinjuku-ku, Tokyo 160-8582, Japan; Department of Cardiology, Tokyo Metropolitan Children's Medical Center, 2-8-29, Musashidai, Fuchu-shi, Tokyo 183-8561, Japan; Department of Cardiovascular Surgery, Keio University School of Medicine, 35 Shinanomachi, Shinjuku-ku, Tokyo 160-8582, Japan

**Keywords:** Premature constriction of ductus arteriosus, Tricuspid atresia, Right ventricle outflow tract obstruction, Case report

## Abstract

**Background:**

Premature constriction of the ductus arteriosus (PCDA) makes management difficult in neonates with congenital heart defects, particularly those with ductal-dependent pulmonary circulation. This report highlights the challenges and management of a neonate diagnosed with tricuspid atresia and severe right ventricular outflow tract obstruction (RVOTO), complicated by PCDA.

**Case summary:**

A male neonate was diagnosed prenatally with tricuspid atresia and severe RVOTO. After birth, his oxygen saturation was around 60%, and no ductus arteriosus was detected. A systemic-to-pulmonary shunt was placed emergently. After surgery, antegrade blood flow from the right ventricular outflow tract was unstable depending on the right ventricular muscle contraction and relaxation, and the antegrade blood flow needed to be occluded. The postoperative course was uneventful after then.

**Discussion:**

This case underscores the complexity of managing neonates with tricuspid atresia, severe RVOTO, and PCDA. Early surgical intervention is critical in stabilizing such patients.

**Learning points**

- Premature constriction of ductus arteriosus (PCDA) leads to significant cyanosis in congenital heart defect, particularly in cases with ductus arteriosus-dependent pulmonary circulation.
- Premature constriction of ductus arteriosus complicated by tricuspid atresia can cause marked right ventricular hypoplasia, which leads to unstable antegrade pulmonary blood flow.
- In neonates with tricuspid atresia and severe right ventricular outflow tract obstruction complicated by PCDA, prompt surgical intervention, such as the placement of a systemic-to-pulmonary shunt and occluding the main pulmonary artery, was crucial for stabilizing the patient’s condition.

## Introduction

Premature constriction of the ductus arteriosus (PCDA) can lead to complete closure of the duct during the foetal period.^1,2^ In congenital heart defect (CHD) with ductal-dependent pulmonary circulation, the ductus arteriosus supplies pulmonary blood flow and is essential for survival. Administration of prostaglandin E1 is usually required to maintain patency of the ductus arteriosus. The reports on the relationship between PCDA and CHD with ductal-dependent pulmonary circulation are scarce, and the clinical strategy has not yet been established.

## Summary figure

**Figure:**
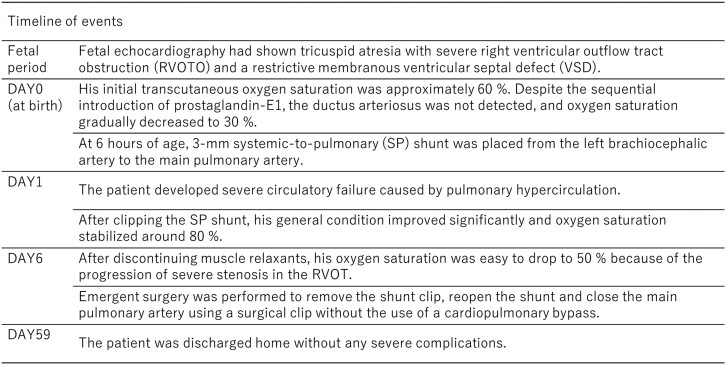


## Case presentation

A male neonate with a birth weight of 2222 g was delivered via caesarean at 37 weeks of gestation. His mother was referred to our institution at 20 weeks gestation because the foetus was suspected to have CHD. Foetal echocardiography had shown tricuspid atresia with severe right ventricular outflow tract obstruction and a restrictive membranous ventricular septal defect (VSD). The ductus arteriosus could not be seen despite repetitive evaluations, and PCDA was suspected.

**Figure 1:**
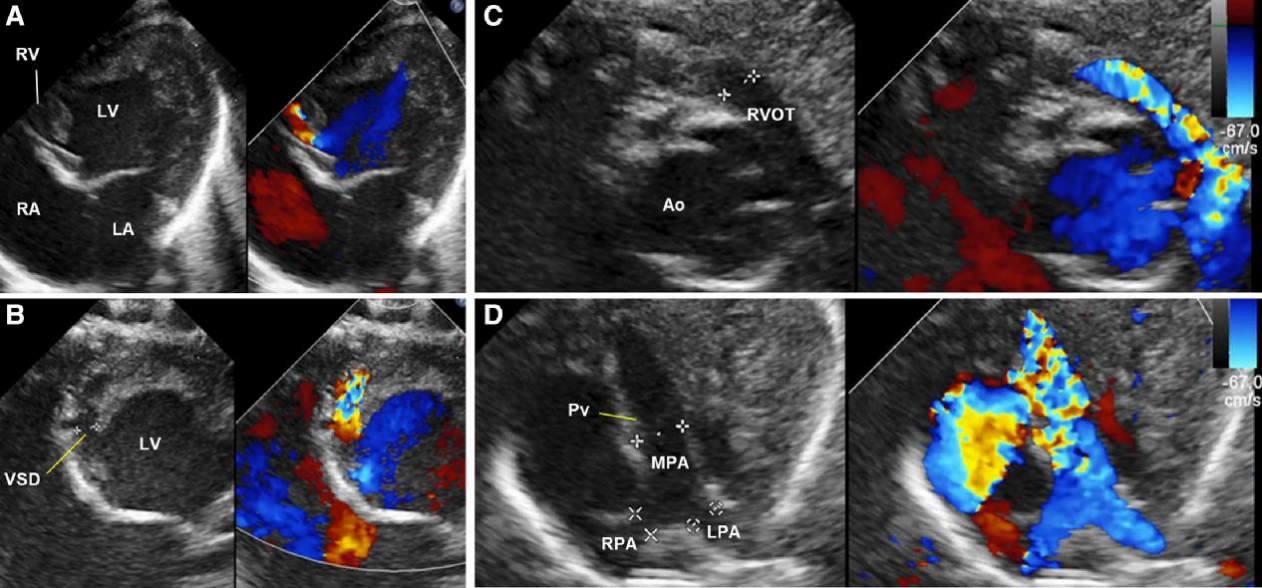
Transthoracic echocardiography after birth. (*A*) Tricuspid atresia and hypoplastic right ventricle, (*B*) a restrictive ventricular septal defect, (*C*) severe right ventricle outflow tract obstruction, and (*D*) hypoplastic main pulmonary artery. No ductus arteriosus could be seen. Ao, aorta; RV, right ventricle; RVOT, right ventricle outflow tract; Pv, pulmonary valve.

After delivery, his initial transcutaneous oxygen saturation was ∼60%. Postnatal transthoracic echocardiography confirmed the prenatal diagnosis, and the ductus arteriosus was not detected. The right ventricular outflow tract (RVOT) exhibited extreme narrowing with a diameter of 2 mm. The main pulmonary artery was also hypoplastic with a diameter of 4 mm (*Z*-score = −3.9) (*Figure 1*). Balloon atrial septostomy was not performed because atrial communication was sufficiently large with a laminar shunt. Despite the sequential introduction of prostaglandin E1, tracheal intubation, ventilatory management, intravenous sedatives, muscle relaxants, and nitric oxide inhalation, the ductus arteriosus was not detected, and oxygen saturation gradually decreased to 30%. At that time, at 6 h of age, a decision was made to take him to the operating room emergently and a 3 mm systemic-to-pulmonary (SP) shunt was placed from the left brachiocephalic artery to the main pulmonary artery. Surgical findings revealed a fibrous ligament supposed of a completely closed ductus arteriosus and the diagnosis of PCDA was confirmed.

**Figure 2:**
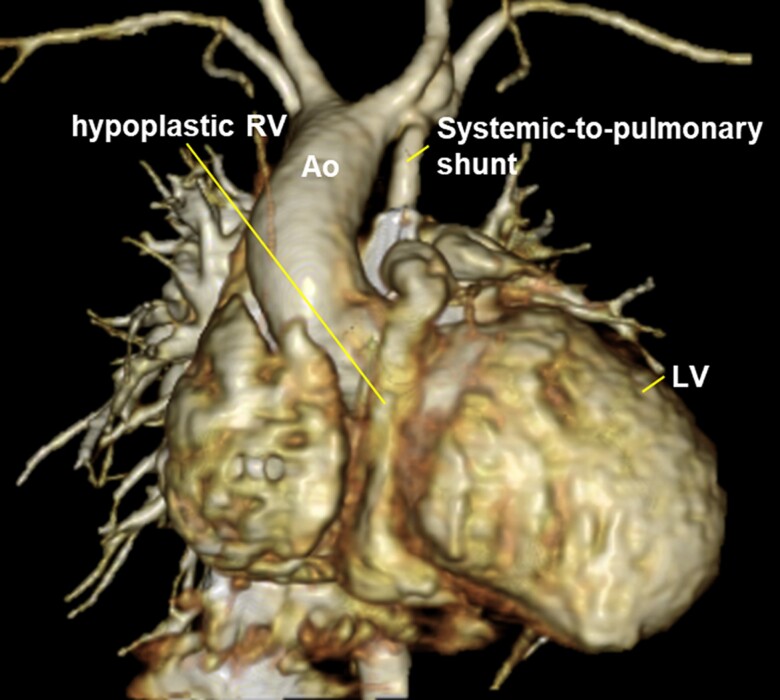
Contrast-enhanced computed tomography showed markedly hypoplastic right ventricle.

On postoperative day 1, the patient developed severe circulatory failure caused by pulmonary hypercirculation leading to a pulmonary-to-systemic flow ratio of 3.0 and an elevated lactate concentration of 11 mmol/L. At that point, a decision was made to clip the SP shunt. After reoperation, his general condition improved significantly, and oxygen saturation stabilized around 80%. However, after discontinuing muscle relaxants on postoperative day 6, his oxygen saturation was easy to drop to 50% because of the progression of severe stenosis in the RVOT. When the patient was placed in the chest–knee position and administered pure oxygen and phenylephrine, his oxygen saturation temporarily increased to around 80%. A decision was made to remove the shunt clip, reopen the shunt, and close the main pulmonary artery using a surgical clip without the use of a cardiopulmonary bypass. The patient underwent this emergent surgery successfully leading to a stable postoperative course. Contrast-enhanced computed tomography showed a markedly hypoplastic right ventricle (RV) (*Figure 2*). Cardiac catheterization performed after surgery revealed a pulmonary-to-systemic flow ratio of 1.4. The patient was discharged home on Day 59 without any severe complications. At the age of 5 months, the patient underwent a successful bidirectional Glenn procedure. Currently, he is doing well and awaiting the Fontan procedure.

## Discussion

This is the first case of a single ventricle with a hypoplastic RV complicated by PCDA requiring a day 0 SP shunt and surgical intervention of RVOT. Premature constriction of the ductus arteriosus has been reported to complicate dextro-transposition of the great arteries.^3^ Systemic-to-pulmonary shunt construction was performed on Day 34 when cyanosis is refractory to therapy such as introduction of prostaglandin E1, balloon atrial septostomy, and NO inhalation. In our case, medical therapy after birth was ineffective because of the complete closure of the ductus arteriosus and the characteristic hypoplastic RV.

Several reports have shown that PCDA results in right ventricular dilatation and severe tricuspid regurgitation due to increased RV afterload during the foetal period.^1,2,4^ In our case, tricuspid atresia and small restrictive VSD resulted in RV hypoplasia due to insufficient RV preload contrary to previous reports. These haemodynamic abnormalities would lead to reduce the right ventricular trabecular component, resulting in a severely hypoplastic RV characterized by a ‘slit-like’ lumen throughout the ventricle.

The postoperative course involved unstable antegrade pulmonary blood flow. In patients with a single ventricle, significant changes in haemodynamics before and after SP shunt placement have been reported.^5,6^ In this case, deep sedation after first surgery caused preventing the narrowing of the ‘slit-like’ RV lumen as well as reducing the pulmonary vascular resistance, which resulted in unacceptable pulmonary hypercirculation. When discontinued deep sedation, the ‘slit-like’ RV lumen, resembling an elongated infundibulum, was dynamically narrowed, leading to repeated ‘anoxic spells’. Haemodynamic stability was achieved by the complete occlusion of the main pulmonary artery.

This case highlights the surgical treatment strategy for the patient with tricuspid atresia with severe RV hypoplasia and PCDA. In cases like this, blocking antegrade blood flow from RVOT and establishing a SP shunt should be considered in the early postnatal period. When the results of prenatal diagnosis are very complex like this case, it is important to plan the birth at a third-level facility with experienced paediatric cardiologists and cardiac surgeons to ensure a good outcome.

## Data Availability

The data underlying this article will be shared upon reasonable request to the corresponding author.

## Lead author biography

Taro Kono graduated from the Faculty of Medicine, Keio University, in 2017. He is currently working as a paediatric cardiology trainee in the Department of Pediatrics, Keio University School of Medicine, Tokyo, Japan.

## Notes

**Consent:** The authors confirm that written consent for submission and publication of this case report, including images and associated text, has been obtained from the patient’s next of kin in line with COPE guidance.

**Funding:** None declared.
